# Complex rice systems to improve rice yield and yield stability in the face of variable weather conditions

**DOI:** 10.1038/s41598-018-32915-z

**Published:** 2018-10-03

**Authors:** Uma Khumairoh, Egbert A. Lantinga, Rogier P. O. Schulte, Didik Suprayogo, Jeroen C. J. Groot

**Affiliations:** 1 Farming Systems Ecology Group, Wageningen University and Research, P.O. Box 430, 6700 AN Wageningen, The Netherlands; 2 Faculty of Agriculture, Brawijaya University, Jalan Veteran, 65145 Malang, Indonesia

## Abstract

Extreme weather events and pest outbreaks decrease rice yields and increase their variability, presenting challenges for the agricultural agenda to increase rice productivity and yield stability in Asia. The integration of azolla, fish and ducks has been shown to create robust systems that maintain high yields under heavy rainfall, but no clear evidence exists that rice yields in these systems are stable across locations and throughout time under divergent weather conditions. We show that the introduction of additional elements into the rice cropping system enhanced the adaptive capacity to extreme weather events across four locations and three cropping cycles. The complex system showed both static and dynamic stability, and had the highest reliability index, thereby outperforming the conventional and organic monoculture systems. The complex rice system design provides a promising example for resilience towards the impacts of climate change on rice production and for safeguarding food security in Asia and beyond.

## Introduction

Climate change is expected to increase the variability in weather conditions and the frequency of extreme weather events such as floods and droughts^1,2^, which will have direct effects on agricultural production worldwide^3^. For example, in Asian rice systems, the anticipated changes in weather patterns could lead to faster development, growth, and spread of weeds and of important crop pests and diseases, presenting a major challenge to food stocks^4–7^. This is of great relevance to Indonesia: although it is the third largest rice producer in the world, demand for rice exceeds production^8,9^, resulting in an insecure national stockpile that remains dependent on imports.

Domestic rice production and food security in Indonesia have recently been affected by extreme weather events that include heavy rainfall in 2013 and in 2016 caused by la Nina, which were interrupted by an el Nino event that prolonged the dry season during 2015. Water logging may decrease rice yields through hypoxia, while heavy rains disturb pollination, fertilization, and grain-filling processes. A longer duration of rainfall reduces light intensity, affecting the drying of grains at the ripening stage. Heavy rainfall may also affect grain quality resulting from shattered, lightweight, blackened or chalky grains^10–12^. In addition, extreme weather events may trigger pest and weed outbreaks. To compensate the resulting decline in production, Indonesian rice imports increased by 44%, from 1.25 million tonnes in 2015 to 1.80 million tonnes in 2016^13^.

Increased use of agro-chemicals aimed at addressing this challenge is not only potentially harmful to the environment and human health, but also increases the dependency of farmers on external inputs^14^. Organic agriculture has been proposed as an option to reduce the negative impacts on the environment and human health, but it is often associated with lower crop yields and more labour-intensive crop management practices, although this is dependent on the type of organic system^15–17^. For example, complex rice systems (CRSs) can be more productive than monoculture systems^18^.

CRSs integrate the cultivation of azolla, fish and ducks into the rice system, and have previously been shown to be a promising approach to ecologically address pest problems and increase rice yields in organic rice production systems^18^. However, to date no clear evidence was available on the stability and applicability of CRSs across locations and throughout time under divergent weather conditions.

In this paper, we measured the yields and assessed yield stability of conventional, organic and complex rice production systems across temporal and spatial scales. Conventional treatments were characterised by the use of artificial fertilizers, pesticides and herbicides, while organic treatments received organic fertilizers and bio-pesticide applications. CRS treatments integrated azolla, fish and ducks into organic rice systems, coupled with growing border plants on 50–100 cm wide ridges surrounding CRS plots. Border plants consisted of green manures and vegetables, which can supply food and feed, and refugia to attract natural enemies. Hence, no pesticides were applied in the CRS treatments. Finally, the potential for the scalability and replicability of the proposed CRSs’ design was discussed in this paper.

## Results

### Rice yield stability and reliability

**Figure 1:**
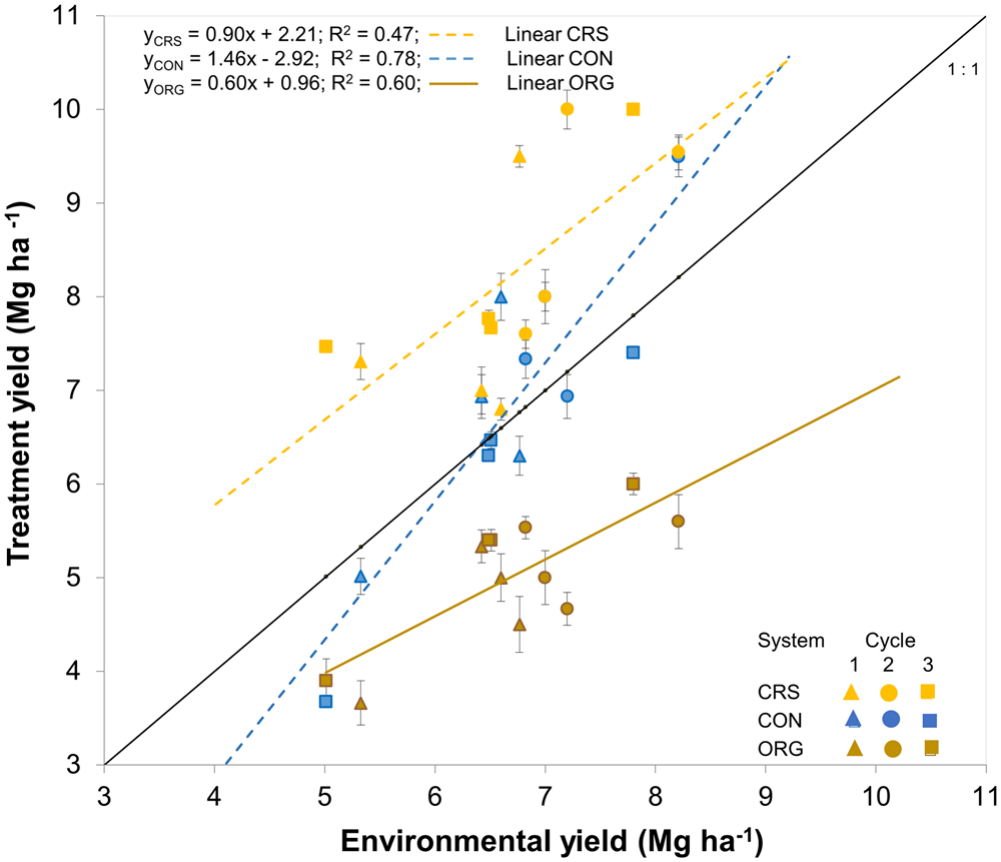
Dynamic yield stability, showing the relationship of rice yields in the three production systems and the environmental mean yields. ORG, organic system; CON, conventional system; CRS, complex rice system. The black line indicates a regression line with slope = 1. The brown line represent the regression line of the yields in the ORG treatment, with a slope significantly lower than 1. The dashed blue and yellow lines represent the regression line of the yields in the CON and CRS treatment respectively, with slopes not significantly deviating from 1.

Rice yields in our experiment were significantly influenced by all experimental factors (locations, cycles, production systems) and their interactions at *P* level < 0.001. The weather conditions in the second cycle were favorable. Thus, rice yields of the conventional production system in the second cycle were higher than the first and third cycle (*F*_(2,33)_ = 7.979, *P* = 0.001; Fig. 1), and ranged from 6.9 to 9.5 Mg ha^−1^. Under the favorable weather conditions of the second cycle, conventional yields were generally higher than in the organic system (*F*_(2,33)_ = 48.366, *P* < 0.001) and ranged from 4.7 to 5.6 Mg ha^−1^. However, the yields of conventional were not higher than second cycle yields in CRS which ranged from 7.6 to 10.0 Mg ha^−1^ (Fig. 1).

The values and statistic assessment of the yield stability are presented in Table 1. Although unfavorable conditions also affected yields in CRSs, these yields were still consistently above the mean yield across all systems (Fig. 1). This resulted in a low CV_i_ in CRS (CV_i_ = 0.14), indicating good static stability. The slope value of CRS was also not significantly different from one in the dynamic stability assessment, demonstrating a stable system.

**Table 1:** Static and dynamic stability analysis of the three rice production systems.

	n	Mean	SE	Slope	t	*P* slope = 1	Intercept	R^2^	CV_i_	I
CON	12	6.8	±0.246	1.46	1.87	0.076	−2.766	0.78	0.22	7.3
ORG	12	5.0	±0.154	0.60	2.58	0.018*	1.053	0.60	0.14	2.6
CRS	12	8.2	±0.304	0.90	0.33	0.746	1.713	0.47	0.14	12.3

CV_i_, coefficient of variance implies the static stability indices, the significant differences (*P* slope) of the three rice production system slopes to the slope of the mean (X = Y) suggest the dynamic stability. I denotes to reliability index (see Methods). CRS, complex rice system; ORG, organic; CON, conventional.

In contrast, the yields in conventional systems were very sensitive to environmental variations, with remarkably high yields in favorable environments and much lower yields under unfavorable conditions. This variability resulted in a high CV_i_ of 0.22, demonstrating unstable yields according to the static concept. However, the yields in the conventional systems were significantly higher than the mean yields of organic systems (F_(12,72)_ = 9.88, P < 0.001) and the slope in the dynamic stability assessment was not significantly different from one, indicating a stable system according to the dynamic concept. Subsequently, the effect of the environmental variations on rice yields in organic systems was small, indicated by a small CV_i_ of 0.14. Yields in organic systems were therefore stable according to the static concept. However, not only were the yields in the organic treatment consistently lower than the mean yield across all systems, also the regression slope was significantly lower than one (*t*_(20)_ = 2.60, *P* = 0.018), indicating dynamic instability.

Besides having both good static and dynamic stability, the CRS had a higher reliability index (I = 12.3) than that in the conventional system (F_(2,6)_ = 75, P < 0.001; I conventional = 7.3). Although the static concept demonstrated that the organic yields were stable, their absolute yields were consistently lower than those in both CRSs and conventional systems, as indicated by a low reliability index (I = 2.6; Table 1).

### Interactions of weather conditions, labor input, rice yield and production system

**Figure 2:**
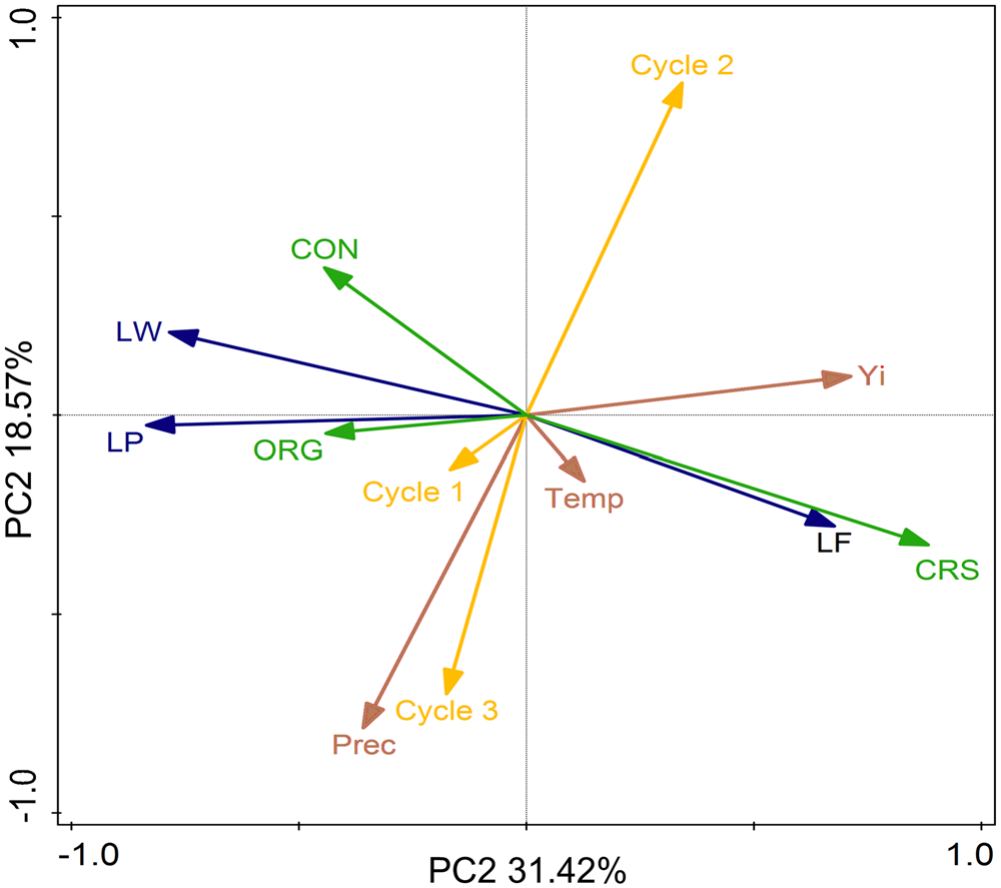
Biplot of the principal component (PC) analysis for the relationships between rice yields (Yi) with the three rice production systems (CON, conventional; ORG, organic; CRS, complex rice systems), labour inputs (LF, labour for fertilization; LP, labour for pest control; LW, labour for weed control), weather conditions (Prec, precipitation; Temp, temperature) and the growing cycle (1–3).

The bi-plot in Fig. 2 shows the interactions between weather conditions, growing cycle, labor input, production system and rice yield. Cycle 2 was associated with high yields and this high yield was strongly linked with CRS. Despite the strong relation with the yield variable, CRSs were negatively associated with labor inputs to control weeds and pests. In contrast, labor input for fertilization was also positively related to CRS. Furthermore, the bi-plot presents a negative correlation between yield and cycles 1 and 3 which were associated with high precipitation levels. This high precipitation in cycle 1 was positively correlated with high labor inputs for pest and weed control in conventional and organic systems.

## Discussion

Notwithstanding the multiplicity of potential yield-reducing factors, we observed that extreme weather events coincided with pest outbreaks, such as plant hoppers and stem borers during the first and third cycle (Supplementary Table S1). These patterns provided a highly variable weather scenario suitable to test resilience of our three systems. Under variable conditions and with the occurrence of extreme events, the most stable system is not necessarily the system with the highest mean yield. In general, CRSs had the highest mean yield and yields were less affected by unfavorable environments than for the other treatments. Likewise, although the yields in organic were lower than in conventional, however, rice yields were reduced less severely than in the conventional treatment during bad cropping cycles. Nevertheless, the strength of this treatment effect varied by location and between cropping cycles.

In Malang, for example, CRS, organic and conventional yields in the first cycle were 23%, 35% and 47% lower, respectively, than in the second cycle (*F*_(2,6)_ = 28,824, *P* = 0.001). Whereas, in the third cycle, reductions of CRS, organic and conventional yields were 22%, 30% and 61%, lower, respectively than in the second cycle (*F*_(2,6)_ = 64.871, *P* < 0.001). The use of bio-pesticides in the organic systems might have been less harmful to natural enemies than by the use of chemical pesticides in conventional rice production. This may help to maintain higher levels of natural enemies (Supplementary Table S2), partly protecting rice plants from pests during unfavourable cycles^19,20^. Besides, organic cropping system obviously eliminates the dependency on agro-chemicals (Supplementary Tables S3–S5), resulting in smaller negative impacts to the environment than conventional systems.

The CRSs appeared to be most resilient to the impacts of extreme weather events on rice yields, by integrating flora and fauna resulting in ecosystem functions of weed and pest suppression and increased nutrient cycling. The feeding and movement behavior of the ducks and fish, combined with the presence of refugia can facilitate natural enemies to control pests and weeds, thereby also reducing labor inputs. The labor input for fertilization in CRS might high, which was allocated to manage green manure biomass, including activities such as cutting, drying and soil incorporation. However, the total labor inputs were much less than those in conventional and organic. The higher the quantity of green manure produced in CRS, resulted in the higher reduction imports of fertilizers (Supplementary Table S3).

In our CRS, duck manure, azolla and sun hemp support the ecosystem function of nutrient cycling (Supplementary Tables S3–S6), which is further accelerated by the ducks and fish activities. Besides, the activities of ducks and fish can increase the oxygen supply which leads to better rice root growth and nitrogen uptake^21–23^. Higher root density increased nutrient uptake to enhance rice yields and might reduce pollutions. Moreover, adding components and outputs to the system such as duck, fish, egg, string bean, corn, taro and papaya could contribute to the stability of whole farm production, especially when one commodity fails during unfavourable weather conditions (Supplementary Table 7).

Our results show that the occurrence of extreme weather events is a deciding factor in reducing rice yields and indeed potentially threatening the stability of global production. The results also revealed that common practices in rice production systems, such as the use of chemicals and extra labor inputs for weeding and pest control, were ineffective to suppress weeds and pests during unfavorable weather conditions to the same degree as in CRSs (Supplementary Tables S4–S6; Supplementary Figure S1). Possible explanations for ineffectiveness of pesticides include (i) rain-induced run-off of pesticides, (ii) alkaline hydrolysis and degradation of pesticides to an inactive form, as induced by fluctuations in temperature and water pH, and (iii) phyto-toxicity of some pesticides induced by high temperatures^24,25^. These effects may be further aggravated in practical situations on smallholder farms, since illiteracy and suboptimal managerial skills of smallholders may cause improper application timing, pesticide mixing and pest identification.

We conclude that CRSs are resilient to the effects of extreme weather events and provide flexible mechanisms to control weeds and pests in unfavourable weather conditions, thus providing more robustness compared to other rice production systems. Finally, the stable and reliable yields of CRSs across strongly contrasting locations in our experiment show that the design is scalable and replicable, warranting out-scaling and prioritizing to other rice producing countries. This can contribute to reducing smallholder risks, while maintaining rice productivity and yield stability to safeguard national food security under climate change.

## Methods

### Study site

The experimental sites were in the Lamongan sub-district of Lamongan (7°08ʹ27.10″S − 112°23ʹ46.79″E), Kepanjen sub-district of Malang (8°09ʹ11.82″S − 112°34ʹ33.32″E), Gandusari sub-district of Blitar (7°59ʹ34.14″S − 112°18ʹ21.79″E) and the Prigen sub-district of Pasuruan (7°41ʹ49.82″S − 112°37ʹ40.44″E). The sub-districts are distributed over a range of altitudes: Lamongan (8 meters above sea level), Kepanjen (326 m.a.s.l), Gandusari (600 m.a.s.l) and Prigen (760 m.a.s.l). The sub-districts cover different soil types and textures: Vertisol-clay, Inceptisol-silty clay, Entisol-sandy and Andisol-sandy clay loam.

The field experiments were undertaken from December 2014 to March 2015 (first cycle), from May to August 2015 (second cycle) and from January to April 2016 (third cycle). We originally planned the third cycle for September to December 2015, but this had to be delayed because of the prolonged dry season in 2015.

### Experimental design and agronomic practices

We compared three different production systems: the complex rice system (CRS), and organic and conventional monocultures. The CRS contained rice (*Oryza sativa*, var. Ciherang), azolla (*A. microphylla*), fish (*Oriochromis nilotichus*), ducks (Anas *Platyrhynchos javanicus*) and border plants on the ridges. The experimental layout was a randomized block design with three replicate blocks at each location and for every cycle. Plots were at least 200 m^2^, surrounded by dikes and separated by a border area with a width of at least seven meters.

Agronomic practices were consistent across treatments, consisted of (i) a 5:1 jajar legowo method, which is a rice-planting method where one out of six plant rows remains empty (to allow better sunlight penetration and facilitate easier plant management), (ii) a trench in the middle of the plots, and (iii) the System of Rice Intensification (SRI) planting method. The practice of SRI consisted of (i) a plant spacing of 25 × 25 cm, which is wider than the common spacing of less than 20 × 20 cm, (ii) transplanting of only 10 days-old single rice seedlings, and (iii) intermittent flooding at early stages of rice growth and was kept flooded from 30 days after transplanting (DAT) until two weeks before the rice was harvested.

### Crops and animal management

The conventional treatments received artificial fertilizers (AF) consisting of 206 kg N per ha, 81 kg P per ha and 105 kg K per ha from *Phonska* (N-P-K: 15-15-15), SP-36 (36% P), KCl (65% K), urea (46% N) and ZA (21% N). *Phonska*, SP-36 and KCl were applied at 5 DAT. Urea and ZA were applied three times at 5, 20, and 50 DAT. Details about the use of pesticides and herbicides are given in Supplementary Tables S3–S6.

Organic plots were treated with duck and green manure without herbicide applications but bio-pesticides were sprayed with frequencies and timing similar to those in conventional plots (Supplementary Tables S3–S6). In CRSs, the percentage of organic fertilizers that was imported from outside the farm was 62% in the first cycle, 29% in the second cycle and 22% in the third cycle. The remaining organic fertilizers were produced on-farm from azolla, sun hemp and animal manure. The description, cultivation and integration methods of the components in the first cycle followed the integration methods described in Khumairoh *et al*.^18^ except for planting spaces, border plants, fences and fertilizers^10^. Another difference in the current experiment was inoculation of azolla was not supported by artificial-phosphor fertilizers.

CRS design was slightly adjusted throughout the rice-growing cycles. Sun hemp (*Crotalaria juncea*), vegetables and fruit were grown two weeks before sowing rice seed. In the second cycle, 90% of planted sun hemp was cut every month after the first cut (45 days old) and incorporated into the soil as green manure or dried for feed. The remaining 10% was left to produce flowers and seeds. Due to the low survival rate of fingerlings in the first and second cycles, leading to low fish yields, fish ponds were deepened and widened. Fingerlings changed to adult fish (four to five months old and about 200 gr per fish) to be released in CRS plots. The adult fish density was 500 fish per hectare that can produce fingerlings between 5–10 days after release. Fingerlings that hatched on the rice field could immediately adapt to the environment, which is a crucial factor for successful fish production.

Duck houses were constructed on the bund surrounded by sun hemp. Straw from previous cropping was heaped next to the duck house and pond to be decomposed and later eaten by fish. The total amount of feed supplied to one duck for the duration of 155 days was approximately 20 kg (Supplementary Table S8). In the first cycle, all duck feed was imported, but in the second and third cycles ducks were fed rice bran and corn from previous cycles. Sun hemp and azolla also provided feed for ducks and fish. The ducks also preyed on wild animals and plants, such as weeds, snails, crabs, tadpoles and insects.

## Measurements and Statistical Analysis

### Weather data

**Figure 3:**
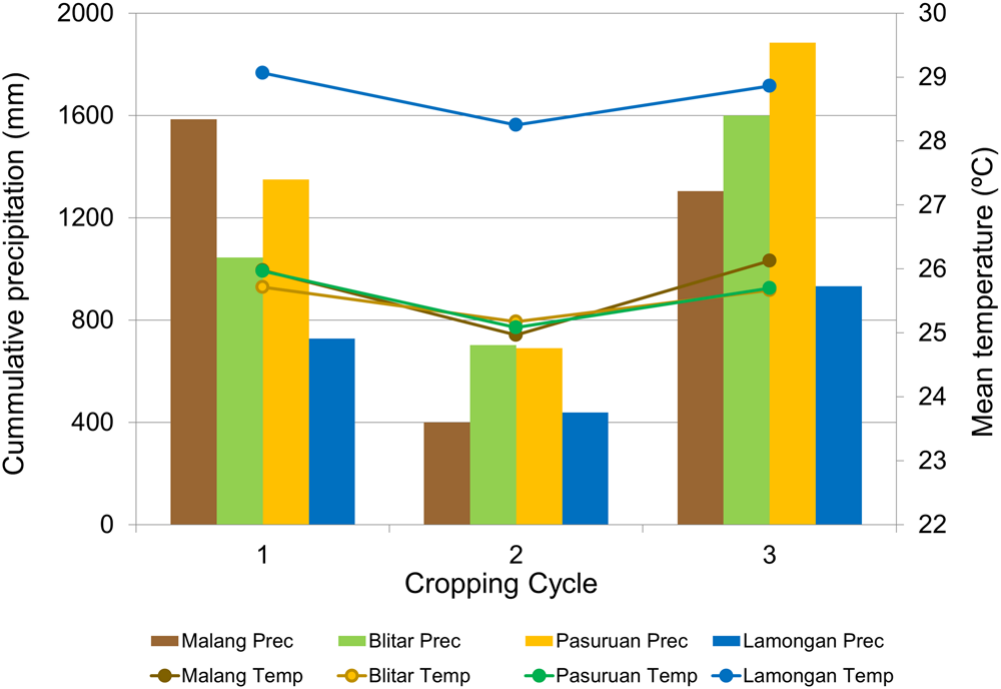
Weather conditions (cumulative precipitation and mean temperature) during the three rice growing cycles of the experiment at the four experimental locations in Regencies of East Java Province, Indonesia.

During the experiment, all weather data were recorded using the weather station installed on each experimental farm. Weather conditions of three rice growing cycles in the experimental sites are provided in Fig. 3. The precipitation amounts during the experiment were significantly different among growing cycles (*F*_(2,11)_ = 7.308, *P* = 0.013), in which growing cycle 2 was the driest, while cycle 3 was the wettest, and cycle 1 was slightly drier than cycle 3.

### Labor inputs

Labor inputs were recorded and are presented in Supplementary Figure S1. In general, artificial fertilizers were applied three times during one rice growing cycle in conventional systems, but only once in organic systems, at three to five days before transplantation of rice plants at the second ploughing stage. Fertilizer application in CRS was similar to that in organic plots but the sources were partly supplied by sun hemp and azolla. Labor inputs for weeding and pests management included: (i) agro-chemical applications and hand weeding in conventional, (ii) spraying bio-pesticides and hand weeding in organic, and (iii) animal management in CRSs.

### Measurement of plants and animals

Rice yield was measured by harvesting 6.25 m^2^ of rice plants in the middle of the plot at harvesting time. Rice grain was removed from the straw and placed in paper bags to be dried in an oven at 80 °C for 48 hours and weighed to obtain the dry matter yield of rice kernels. Fresh weight of fish, ducks and eggs were determined at the end of each cycle. A sample of sun hemp plants was oven dried at 80 °C for 48 hours and weighed to record the dry matter mass. Kangkung (*Ipomoea aquatic*) was harvested three times during one rice-growing cycle.

### Pests and natural enemies assessment

Insects and natural enemies were collected using fine nylon cloth sweep net to assess their abundance. The sweep net was 35 cm in diameter and had a 65 cm length handle. Sweeping was done around 180° from the plant canopy level to the basal region of the plants. Ten nets were swept in each plot at 90 DAT and the insects were kept in a clear bottle containing 75% alcohol. Snails were collected from 1 m^2^ of surface soil, washed and counted.

### Statistical analysis

We tested the normal distribution of data using the Shapiro-Wilk, Kolmogorov-Smirnov tests, and Skewness and Kurtosis tests. We used log10 and sqrt transformations where necessary. Analysis of variance (ANOVA) was performed to test the experimental treatment effects followed by Tukey’s and LSD post-hoc tests to establish significant differences between treatments. Statistical tests were conducted with SPSS 23 software package (SPSS Inc., Chicago, Illinois, USA). We visualized interactions between rice yields, environment, rice production systems and labor inputs using the biplot function in the CANOCO 5 software for Windows.

We used static and dynamic stability analysis approaches in our study. A static approach evaluates rice yield stability across all environments (location*cycle), using the coefficient of variation (CV_i_). Low values of CV_i_ are seen as desirable, implying a constant yield across targeted environments^26,27^. A dynamic stability approach was assessed using Finlay and Wilkinson’s regression model (1963)^28,29^. The slope of each rice production system was tested against the slope of the overall mean regression line (*b* = 1) using a t-test, assuming greatest stability for slopes closest to this line^30–32^. To generate a useful comparison to make recommendations, we calculated a yield reliability index (I) that combined yield stability and mean yield^31–33^. The calculation was adopted from Kataoko that takes into account the “riskiness” of production systems^34,35^, which is specified as:

$${{\rm{I}}}_{{\rm{i}}}={{\rm{m}}}_{{\rm{i}}}-{{\rm{Z}}}_{({\rm{p}})}^{\ast }Si,$$

where m_i_ is the mean yield of production system *i*, Z_(p)_ is the percentile from the standard normal distribution that reached the value *P*. We defined *P* as a risk of the yield in production system *i* to fall below the mean yield of all production systems, ranging from Z_(100%)_ = 3.4 to Z_(0%)_ = −3.4.*Si* is the square root of the environmental variance that is expressed as:

$$Si=\sqrt{\sum _{i=1}^{n}{(xi-x)}^{2}/(n-1)}$$

where x_i_ is the yield of production system *i* in a certain environment, x is the mean yield of production system *i* across all environment, and n is the number of environmental combinations.

## Electronic supplementary material

Supplementary Information

Supplementary Dataset 2
