# Supplementary material for: Complex rice systems to improve rice yield and yield stability in the face of variable weather conditions
Source: Sci Rep. 2018 Oct 3;8:14746. doi: 10.1038/s41598-018-32915-z (PMC6170462; doi:10.1038/s41598-018-32915-z)
Supplement: Supplementary file 1 — Supplementary Information [file 41598_2018_32915_MOESM1_ESM.pdf]

Supplementary information

Complex rice systems to improve rice yield and yield stability in the face of variable weather conditions

Uma Khumairoh*[1,2], Egbert A. Lantinga[1], Rogier P.O. Schulte[1], Didik Suprayogo[2] and Jeroen C.J. Groot[1]

[1]Farming Systems Ecology Group, Wageningen University and Research, P.O. Box 430, 6700 AN Wageningen, The Netherlands.

[2]*Faculty of Agriculture, Brawijaya University, Jalan Veteran 65145 Malang, Indonesia.

Tel: +62 (341) 551665; Fax: +62 (341) 550011. E-mail: uma.khumairoh@wur.nl / uma.kh@ub.ac.id

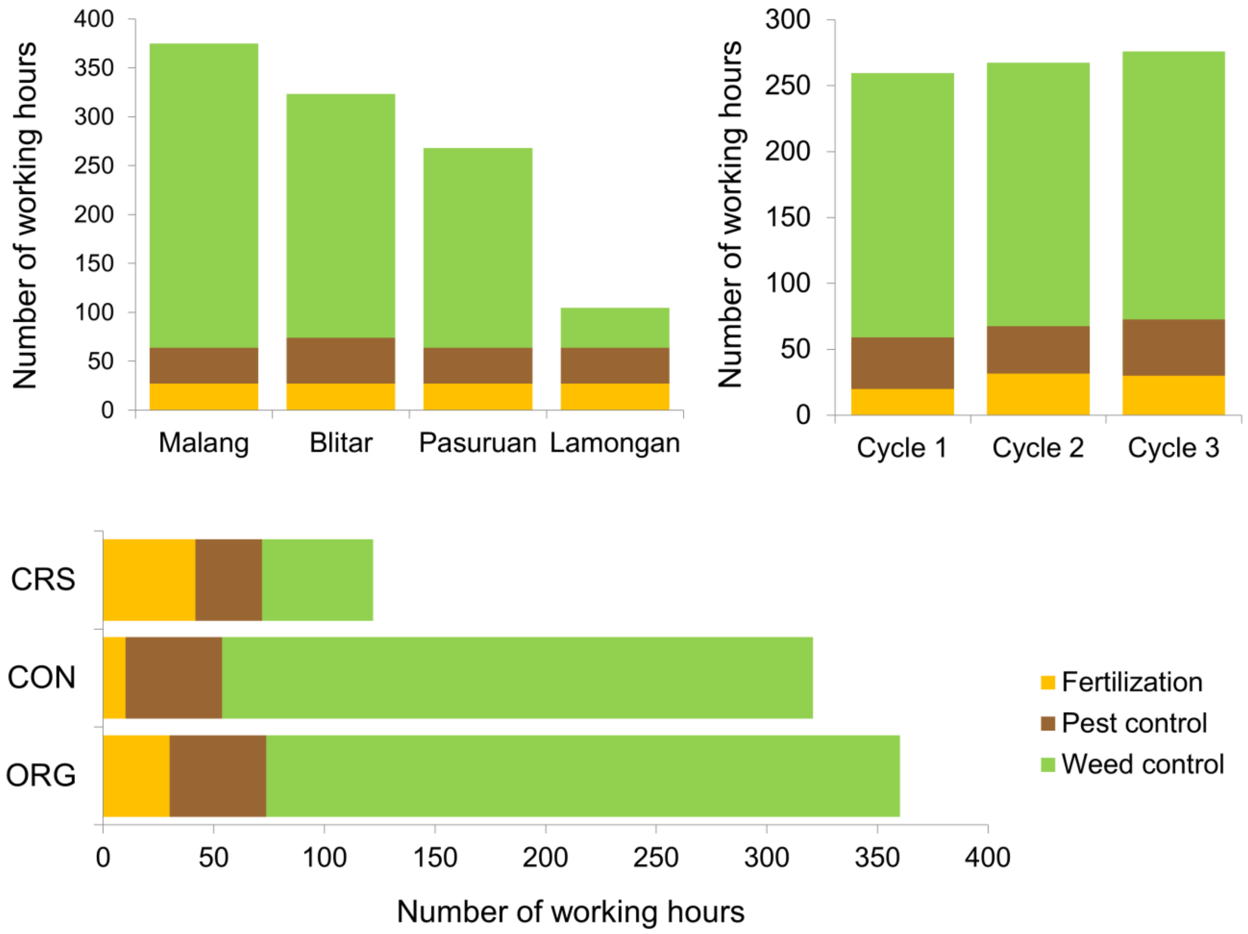

Figure S1. Labour inputs for fertilization, pest and weed control averaged for (a) the four locations of our experimental sites in East Java, (b) the first, second and third rice cultivation cycle, and (c) the three rice production systems; CRS, complex rice system; CON, conventional; ORG, organic. The different colors indicate tasks of fertilizer application, pest control and weed control.

Table S1. Major pest abundance in rice cultivation experiments during three cultivation cycles in four locations in East Java, Indonesia. The treatments represent alternative rice cultivation systems of CON: conventional, ORG: organic and CRS: complex rice system.

		**Stem borers**	Leaf folder	Worl maggot	**Brown plant hopper**	Rice bug	Case worm	Grass hopper	Green hopper	Snails
Location	Lamongan	4.37 b	1.11 a	1.37 b	9.74 c	3.89 b	0.96 a	4.11 b	2 b	14.81 c
	Pasuruan	6.44 c	3.93 c	0.81 a	4.15 b	7.89 c	1.52 bc	5.81 c	2.7 c	9.74 b
	Malang	3.81 a	3.67 bc	2.03 c	1.44 a	2 a	1.7 c	3.22 a	1. 33 a	10.30 b
	Blitar	4 ab	3.19 b	2.78 d	4.70 b	10.93 d	1.07 a	3.11 a	2.6 c	0 a
	df	(3,72)	(3,72)	(3,72)	(3,72)	(3,72)	(3,72)	(3,72)	(3,72)	(3,72)
	F	110.111	52.93	53.769	176.121	238.862	7.28	37.32	18.49	345.865
	P	< 0.001	< 0.001	< 0.001	< 0.001	< 0.001	< 0.001	< 0.001	< 0.001	< 0.001
Cycle	1	0 a	0.94 a	1.06 b	1.57 b	3.33 c	1.19 a	3.72 b	2.36 a	8.44 a
	2	0.36 a	0.44 a	0.56 a	1.16 a	2.42 a	0.97 a	2.67 a	2.03 a	8.17 a
	3	13.61 b	7.53 b	3.64 c	2.4 c	9.28 b	1.78 b	5.81 c	2.06 a	9.53 b
	df	(2,72)	(2,72)	(2,72)	(2,72)	(2,72)	(2,72)	(2,72)	(2,72)	(2,72)
	F	5997.41	675.1	273	195.847	313.505	13.46	81.303	2.18	6.131
	P	< 0.001	< 0.001	< 0.001	< 0.001	< 0.001	< 0.001	< 0.001	< 0.001	0.003
Treatment	CON	6.25 c	3.75 b	1.94 b	8.56 c	7.69 b	1.42 b	5.06 b	3.39 c	11.06 b
	ORG	5.53 b	3.44 b	1.97 b	5.36 b	8.19 b	1.81 c	4.75 b	2.17 b	11.56 b
	CRS	2.19 a	1.72 a	1.33 a	1.11 a	2.64 a	0.72 a	2.39 a	0.89 a	3.53 a
	df	(2,72)	(2,72)	(2,72)	(2,72)	(2,72)	(2,72)	(2,72)	(2,72)	(2,72)
	F	466.564	51.63	13	524.39	187.367	23.42	67.877	99.607	239.765
	P	< 0.001	< 0.001	< 0.001	< 0.001	< 0.001	< 0.001	< 0.001	< 0.001	< 0.001

Table S2. Natural enemy abundance in rice cultivation experiments during three cultivation cycles in four locations in East Java, Indonesia. The treatments represent alternative rice cultivation systems of CON: conventional, ORG: organic and CRS: complex rice system.

		CON	ORG	CRS	df	F	P
Cycle 1	Lamongan	19±4.04a	56.67±1.53b	51.3±2.6b	(2,6)	50.41	<0.001
	Pasuruan	9.3±1.76a	36±2.64b	30.7±2.8b	(2,6)	32.78	0.001
	Malang	23.3±3.53a	36±9.16ab	55±2b	(2,6)	17.15	0.003
	Blitar	11±1.15a	22±1b	19.7±1.2b	(2,6)	26.68	0.001
	**Mean**	**15.7±3.3 a**	**37.4±6.9 b**	**39.2±8.4 b**	(2,33)	13.014	<0.001
Cycle 2	Lamongan	22±1.15a	50.67±9.5b	51.7±1.2b	(2,6)	25.89	0.001
	Pasuruan	11±1.73a	42.3±0.88b	39.3±1.2b	(2,6)	171.72	<0.001
	Malang	22.7±2.18a	33±3.6ab	35±3.21b	(2,6)	6.7	0.029
	Blitar	12±0.58a	38±0.58c	28.3±1.67b	(2,6)	150.42	<0.001
	**Mean**	**17±3 a**	**41±3.7 b**	**38.6±5 b**	(2,33)	33,26	<0.001
Cycle 3	Lamongan	15±2.88a	51.67±1.78b	54±2b	(2,6)	83.89	<0.001
	Pasuruan	12±1.53a	61±3b	56±1.53bb	(2,6)	155.79	<0.001
	Malang	12.7±2.96a	54±2b	48±2.64b	(2,6)	87.46	<0.001
	Blitar	9.3±1.2a	23.3±1.53b	20.3±1.2b	(2,6)	44.46	<0.001
	**Mean**	**12.3±1.2 a**	**47.5±8.3 b**	**44.6±8.3 b**	(2,33)	28,6	<0.001

Table S3. Sources and amount of fertilizers application in rice cultivation experiments during three cultivation cycles in four locations in East Java, Indonesia, in three alternative rice cultivation systems of conventional, organic and complex rice system.

Cycle	Conventional					Organic					Complex rice system				
	Sources	Amount (kg)	N	P	K	Sources	Amount (kg)	N	P	K	Sources	Amount (kg)	N	P	K
											**External**				
**1**	Urea (46% N)	350	161			Compost+GM	6750				Compost+GM	4200			
	ZA (21% N)	0				3 % N		203			3 % N		126		
	Phonska					1% P_2O_5			68		1% P_2O_5			42	
	15-15-15:N-P-K	300	45	45	45	0.9% K_2O				61	0.9% K_2O				38
	SP-36 (36% P2O5)	100		36							**Internal**				
	KCL (65% K_2O)	100			60						Duck's manure	5930			
											1,3 % N		77		
											1,4% P_2O_5			85	
											1 % K_2O				59
	Total		**206**	**81**	**105**		**6750**	**203**	**68**	**61**		**10130**	**203**	**127**	**97**
**2**	Urea (46% N)	225	104			Compost+GM	6750				**External**				
	ZA (21% N)	275	58			3 % N		203			Compost+GM	2000			
	Phonska					1% P_2O_5			68		3 % N		60		
	15-15-15:N-P-K	300	45	45	45	0.9% K_2O				61	1% P_2O_5			20	
	SP-36 (36% P_2O_5)	100		36							0.9% K_2O				18
	KCL (65% K_2O)	100			60						**Internal**				
											Duck's manure	5930			
											1,7 % N		101		
											1,5% P_2O_5			89	
											1 % K_2O				59
											Sun hemp	1500			
											3,5 % N		45		

											0,46% P_2O_5			7	
											1,47% K_2O				22
	Total		**206**	**81**	**105**		**6750**	**203**	**68**	**61**		**9430**	**206**	**116**	**99**
**3**	Urea (46% N)	350	161			Compost+GM	6750				**External**				
	ZA (21% N)	0				3% N		203			Compost+GM	1500			
	Phonska					1% P_2O_5			68		3 % N		45		
	15-15-15:N-P-K	300	45	45	45	0.9% K_2O				61	1% P_2O_5			15	
	SP-36 (36% P_2O_5)	100		36							0.9% K_2O				14
	KCL (65% K_2O)	100			60						**Internal**				
											Duck's manure	5930			
											2 % N		119		
											1,5% P_2O_5			89	
											1 % K_2O				59
											Sun hemp	1200			
											3,5 % N		43		
											0,46% P_2O_5			6	
											1,47% K_2O				18
	Total		**206**	**81**	**105**		**6750**	**203**	**68**	**61**		**8630**	**206**	**109**	**90**

Table S4. Types and amount of active ingredient of herbicide application in conventional plots at four locations for three rice cultivation cycles.

Site	Name of Active Ingredient	Amount (g ha^{-1}) Cycle 1	Cycle 2	Cycle 3
**Lamongan**	2,4-D isopropil amina	1038	692	1038
Total		1038	692	1038
**Pasuruan**	2.4DMA	169	149	176
	Metil Metsulfuron	22	25	29
	Metil Klorimuron	22	25	29
	Triasulfurom	23	16	27
Total		236	214	261
**Malang**	Paraquat	275	275	275
	Ipa Glifosat	243	243	486
	2.4DMA	635	568	568
	Metil Metsulfuron	34	23	23
	Metil Klorimuron	34	23	23
	Triasulfurom	23	23	23
Total		1243	1153	1396
**Blitar**	Ipa Glifosat	486	243	486
	2.4 DMA 865 gr/L (DMA, 0,5 L)	433	433	433
	2.4DMA	635	568	635
	Metil Metsulfuron	34	23	34
	Metil Klorimuron	34	23	34
	Triasulfurom	23	23	23
Total		1644	1311	1644

Table S5. Active ingredient types and amount of pesticides application in conventional plots in four experimental sites at three rice cultivation cycles.

District	Name of Active Ingredient	Amount (g ha^{-1}) Cycle 1	Cycle 2	Cycle 3
**Lamongan**	Difenokonazol	173.0	173.0	173.0
	Azoxistrobin	100.0	100.0	100.0
	Total	273	273	273
**Pasuruan**	Difenokonazol	125.0	125.0	187.5
	Fopronil	25.0	25.0	37.5
	Deltamethrin	18.8	18.8	18.8
	Alfametrin	15.0	15.0	15.0
	Klorantraniliprol	40.0	40.0	40.0
	Total	224	224	299
**Malang**	Karbofuran	240.0	240.0	240.0
	Difenokonazol	125.0	125.0	188.0
	Fopronil	25.0	25.0	37.5
	Deltamethrin	12.5	12.5	18.8
	Endosulfan	175.0	175.0	262.5
	Kumatetralil	7.5	1.5	7.5
	Total	585	579	753
**Blitar**	Klorotalonil	375.0	375.0	562.5
	Difenokonazol	226.0	226.0	226.0
	Azoxistrobin	61.5	61.5	61.5
	Permetrin	100.0	100.0	100.0
	Klorotaloni	10.0	10.0	10.0
	Total	773	773	960

Table S6. Summary of farm works performed for fertilization, weed and pest management in the three rice production systems, CON: conventional, ORG: organic and CRS: complex rice system.

	CON	ORG	CRS	
Fertilization				
Sources	External	External	External	Internal
Form	AF	AGM	AGM	A, Cj, AE
Application	HS	HP	HP	CI & AA
Frequency	3	1	1	3 & AT
Done by	FW	FW	FW	FW & A
Weed management				
Sources	External	External	External	Internal
Form	H	M	M	M
Application	HS & HW	HW	HW & FD	AA
Frequency	3	3	1	AT
Done by	FM	FM	FM	FM & A
Pest management				
Sources	External	External	External	Internal
Form	P	N	N	N
Application	SP	SB	FD	AA & HM
Frequency	7-9	7-9	AT	AT
Done by	FM	FM	FM	A & N

Form of fertilizers: AF, artificial fertilizers; AGM, animal and green manure; A, azolla; Cj, *Crotalaria juncea* (sun hemp); AE, animal excreta. Form of weed management: H, herbicides; M, manual. Form of pest management: P, pesticides; N, natural. Application of fertilizers by: HS, hand spreading; HP, heaping and ploughing; CI, cut and incorporation; AA, animal activities. Application of weed management: HS, herbicides spraying; HW, hand weeding; FD, feeding ducks; AA, animal activities. Application of pest management: SP, spraying pesticides; SB, spraying biopesticides; FD, feeding

ducks; AA, animal activities; HM, habitat management. Works done by: FM, farm workers; AA, animals; N, nature.

Table S7. Alternative products in complex rice systems during three cultivation cycles in four locations in East Java, Indonesia.

	Lamongan			Pasuruan			Malang			Blitar		
	Cycle			Cycle			Cycle			Cycle		
	1	2	3	1	2	3	1	2	3	1	2	3
Animal products												
Fish	0.57	0.64	1.25	0.34	0.72	1.7	0.29	0.41	2.65	0.71	0.76	0.76
Ducks	0.72	0.72	0.72	0.7	0.73	0.7	0.71	0.74	0.72	0.71	0.71	0.74
Eggs	0.07	0.07	0.07	0.06	0.07	0.07	0.07	0.07	0.07	0.05	0.07	0.07
Crop products												
Bean	0.74	0.61		0.45	0.43	0.46	0.68	0.72	0.41	0.51	0.46	0.45
Tomatoes					0.54						0.57	
Taro						0.78						
Sweet Potatoes												1.45
Kangkung									2.35			
Pak choy									0.75			
Fruit (Papaya)									0.24			
Shallot		0.13										
Cucumber		1	0.95									
Gourd			1.25									
Green manure/ feed												
Sun hemp	0.69	2.08	1.61	0.67	2.14	1.53	0.66	2.08	1.47	0.65	2.02	1.44

Table S8. Feed sources and total amount of feed for ducks from 0-154 days old in complex rice systems during three cultivation cycles in four locations in East Java, Indonesia.

Sources	Amount of feed per duck (Kg)	Total feed for 400 ducks (Kg)		
		External	Internal	Total
Rice bran	8.75	300	3200	3500
Dried rice	2.24	896		896
Corn	2.17	268	600	868
Dried fish	0.21	84		84
Sun hemp	5.9		2360	2360
Azolla	2.4		960	960
**Total (Kg)**	21.67	1548	7120	8668
Rice bran	8.75	300	3200	3500
Dried rice	2.24	896		896
Corn	2.17	268	600	868
Dried fish	0.21	84		84
Sun hemp	5.9		2360	2360
Azolla	2.4		960	960
**Total**	21.67	1548	7120	8668
